# The Role of PI3K/AKT/mTOR Signaling in Tumor Radioresistance and Advances in Inhibitor Research

**DOI:** 10.3390/ijms26146853

**Published:** 2025-07-17

**Authors:** Jian Zhan, Manfred Jücker

**Affiliations:** Institute of Biochemistry and Signal Transduction, University Medical Center Hamburg-Eppendorf, Martinistraße 52, 20246 Hamburg, Germany; jianzhan19960827@gmail.com

**Keywords:** PI3K, AKT, mTOR, radioresistance

## Abstract

Cancer is a major threat to human health, and radiotherapy is a key treatment method. However, its effectiveness is often limited by tumor radioresistance. The PI3K/AKT/mTOR signaling pathway is commonly dysregulated in cancers and plays a significant role in radioresistance, though its exact mechanisms remain unclear. This review discusses how this pathway regulates tumor radioresistance and highlights recent progress in the development of related inhibitors in preclinical and clinical studies. These findings aim to guide clinical treatment strategies and provide new approaches to overcoming radioresistance.

## 1. Introduction

In recent years, the incidence of cancer has been steadily rising, making it one of the leading threats to human health. According to the American Cancer Society’s 2024 statistics, cancer has become the primary cause of death for individuals under the age of 85, with over 2 million new cases and 610,000 deaths expected in 2024 [1]. Radiotherapy is a major treatment modality for malignant tumors, with approximately 50% of patients requiring radiotherapy during their treatment [2]. Radiotherapy utilizes high-energy radiation to target tumor tissues, damage cellular DNA, and inhibit cell proliferation, thereby killing tumor cells and controlling tumor growth. However, the resistance of tumor cells to radiotherapy significantly limits its therapeutic efficacy [3,4,5]. To address this challenge, research efforts have focused on two main strategies: increasing radiation doses and decreasing tumor radioresistance. Advanced technologies such as intensity-modulated radiotherapy (IMRT) and stereotactic body radiotherapy (SBRT) allow higher radiation doses to be delivered while minimizing damage to normal tissues, achieving promising outcomes in diseases like liver and lung cancers [6,7]. Additionally, decreasing radioresistance requires an in-depth exploration of the molecular differences between radiosensitive and radioresistant tumor cells, as well as identifying critical targets to achieve radiosensitization by inhibiting relevant signaling pathways [8].

With the advancements in targeted therapy, radiotherapy is no longer limited to local treatment but has evolved into a systemic therapeutic approach by being combined with targeted therapies, thereby increasing treatment efficacy. In addition, the occurrence of abscopal effects has been described where radiotherapy-induced regression or remission occurs in unirradiated distant metastatic lesions when a local tumor site is irradiated [9,10]. The PI3K/AKT/mTOR signaling pathway plays a central role in regulating cell proliferation, survival, metabolism, and transcription and is a critical network in tumor development and progression [11,12,13]. Studies have shown that the overactivation of this pathway can improve tumor radioresistance through mechanisms such as promoting DNA damage repair, inhibiting apoptosis, and regulating the cell cycle [14]. In recent years, inhibitors targeting the PI3K/AKT/mTOR pathway have received significant attention, providing new directions to enhance the effectiveness of radiotherapy and demonstrating potential in multiple clinical trials [15]. This review focuses on the role and molecular mechanisms of the PI3K/AKT/mTOR signaling pathway in tumor radioresistance, summarizes the progress in research on related inhibitors, and evaluates their potential applications in clinical radiotherapy. Through this review, we aim to provide new insights and therapeutic strategies for overcoming tumor radioresistance.

## 2. The Role of the PI3K/AKT/mTOR Pathway in Tumor Radioresistance

**Figure 1:**
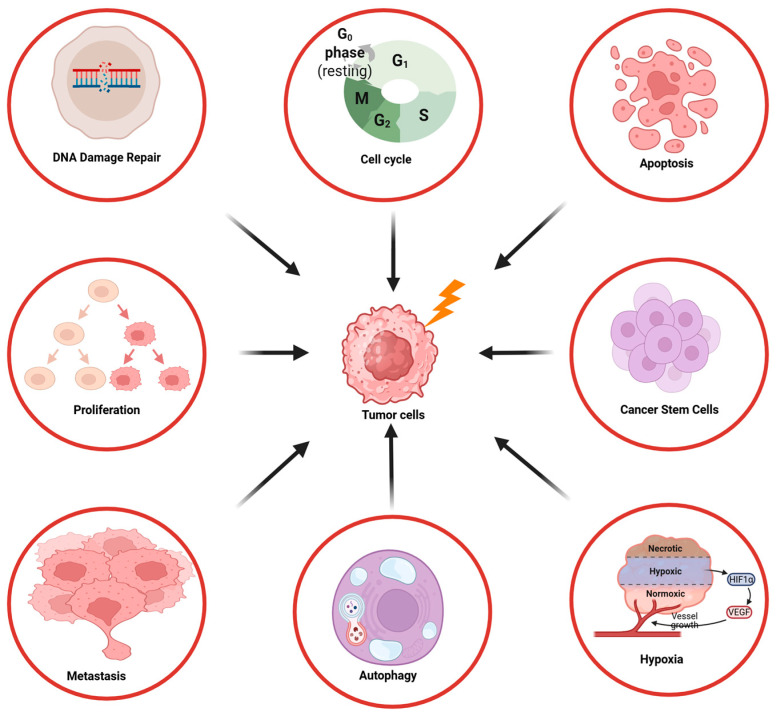
Overview of the influence of the PI3K/AKT/mTOR signaling pathway on the radioresistance of tumor cells.

The PI3K/AKT/mTOR pathway plays an important role in various processes of irradiated tumor cells, including DNA repair, cell cycle regulation, proliferation, apoptosis, autophagy, hypoxia, cancer stem cell self-renewal, invasion, and metastasis, as described in the next chapters in more detail (Figure 1).

### 2.1. DNA Damage Repair

DNA double-strand breaks (DSBs) are among the most severe forms of damage caused by ionizing radiation and can trigger a series of DNA damage responses (DDRs), including damage sensing, signal transduction, cell cycle arrest, and the activation of DNA repair mechanisms [16]. The ability of cells to successfully repair radiation-induced DNA damage largely determines their fate, and the dysregulation of DNA repair pathways is closely associated with tumor radioresistance. Ionizing radiation generates approximately 10,000 DNA base lesions, 1000 single-strand breaks (SSBs), and 40 DSBs per gray of radiation dose [17]. Various types of DNA damage, including adducts, intrastrand and interstrand crosslinks, SSBs, and DSBs, activate distinct repair mechanisms. These primarily include base excision repair, nucleotide excision repair, mismatch repair, homologous recombination (HR), and non-homologous end joining (NHEJ) [18].

The PI3K/AKT/mTOR signaling pathway plays a crucial role in DNA repair by regulating the NHEJ and HR repair pathways. Aberrant activation of this pathway significantly enhances DNA repair capacity, thereby reducing the antitumor effects of radiotherapy. Studies have shown that PI3K/mTOR inhibitors, such as PKI-587 and NVP-BEZ235, can markedly disrupt NHEJ repair efficiency, primarily by modulating the activity of the DNA-dependent protein kinase catalytic subunit (DNA-PKcs). The NHEJ process begins with the recognition of DSBs by the Ku70/Ku80 complex, followed by the recruitment and activation of the DNA-PKcs to facilitate the repair process. The phosphorylation of DNA-PKcs at the Serine 2056 (S2056) site is a critical step in assembling the repair complex. Hyperactivation of the PI3K/mTOR pathway enhances DNA-PKcs phosphorylation, accelerating DSB repair and increasing tumor cell resistance to radiation-induced damage. Conversely, PI3K/mTOR inhibitors significantly reduce DNA-PKcs (S2056) phosphorylation, weaken the binding efficiency of Ku70/Ku80 to broken DNA, and delay repair initiation. This is evidenced by the prolonged dissipation of γ-H2AX foci, indicating reduced NHEJ repair efficiency and decreased tumor radioresistance [19,20]. Furthermore, Apatinib delays γ-H2AX foci disappearance by suppressing the PI3K/AKT signaling pathway, thereby reducing HR efficiency. It also impairs DDR by inhibiting AKT phosphorylation, leading to decreased recruitment and the activation of repair proteins like Rad51 Recombinase (Rad51) [21].

In the HR repair process, mTOR signaling enhances repair efficiency by regulating Fanconi anemia complementation group D2 (FANCD2) expression. FANCD2 is a key factor in the Fanconi anemia pathway, and its function depends on the activation of Ataxia telangiectasia mutated (ATM)/Chk2 signaling. Studies have shown that mTOR inhibitors, such as AZD8055, can downregulate FANCD2 expression, reduce ATM/Chk2 activity, and weaken the repair capacity of the Fanconi anemia pathway, thereby exacerbating DNA damage accumulation. Additionally, FANCD2 downregulation further suppresses Ataxia telangiectasia and Rad3-related (ATR)/Chk1 signaling, limiting tumor cells’ ability to respond to radiation-induced damage [22].

### 2.2. Cell Cycle

The cell cycle consists of four phases: the mitotic (M) phase, the DNA synthesis (S) phase, and the gap phases G1 and G2 [23]. The radioresistance of cells is closely related to their cycle phase, with cells being most radiosensitive during the G2 and M phases, less sensitive during the G1 phase, and least sensitive in the late S phase [24].

In nasopharyngeal carcinoma, the overexpression of Chromosome 2 open reading frame 40 (C2orf40) regulates the PI3K/AKT/mTOR pathway, significantly reducing the protein expression levels of key cell cycle regulators, including Cyclin-dependent kinase 1 (CDK1), Cyclin E1, and Cyclin B1. It also inhibits the phosphorylation of CDK1 and Retinoblastoma protein (Rb), leading to G2/M phase cell cycle arrest. This G2/M arrest not only reduces tumor cell proliferation but also delays the repair of radiation-induced DNA damage [25]. In esophageal squamous cell carcinoma, high family with sequence similarity 135, member B (FAM135B) expression activates the PI3K/AKT/mTOR pathway, shortening the duration of G2/M arrest. The knockdown of FAM135B significantly decreases p-AKT and p-mTOR levels, reduces CDK1 phosphorylation, and weakens the activities of Chk2 and Cyclin B1, thereby delaying the repair of DSBs [26]. In gastric cancer models, high expression of the adenosine A2a receptor (A2aR) enhances AKT and mTOR activity, accelerating DSB repair and improving tumor cell survival. Additionally, A2aR overexpression induces G1 phase arrest and significantly upregulates stem cell markers such as Nanog, octamer-binding transcription factor 4 (OCT-4), and SRY-box transcription factor 2 (SOX-2), providing tumor cells with enhanced proliferative and repair capabilities. Treatment with an A2aR-specific antagonist effectively suppresses PI3K/AKT/mTOR signaling activation and reduces the occurrence of G1 phase arrest [27].

### 2.3. Cell Proliferation and Apoptosis

The proliferation and apoptosis of tumor cells are key characteristics of tumors and important indicators for evaluating tumor malignancy and behavior. In glioblastoma (GBM) models, studies have shown that the overexpression of miR-4524b-5p downregulates the PI3K/AKT/mTOR signaling pathway, inhibiting the expression of the proliferation-related factor Ki-67. Furthermore, miR-4524b-5p activates the mitochondrial-mediated apoptosis pathway, characterized by the upregulation of pro-apoptotic proteins Bcl-2-associated X protein (Bax) and cleaved caspase-3 and a significant downregulation of the anti-apoptotic protein B-cell lymphoma 2 (Bcl-2). These changes markedly enhance radiotherapy-induced apoptosis [28]. Similar molecular alterations have been observed in other tumor models. In prostate cancer, high expression of Apelin Receptor (APLNR) activates the PI3K/AKT/mTOR signaling pathway, increasing levels of p-PI3K, p-AKT, and p-mTOR. This activation further upregulates Bcl-2 expression, inhibits Bax activity, and reduces cleaved caspase-3 levels. These changes diminish tumor cells’ response to radiation-induced apoptosis, thereby increasing radioresistance [29]. In non-small-cell lung cancer (NSCLC) models, the overexpression of Sirtuin 6 (SIRT6) downregulates p-PI3K, p-AKT, and p-mTOR levels, significantly inhibiting the expression of the proliferation marker Ki-67. Additionally, SIRT6 upregulates Bax and cleaved caspase-3 levels while suppressing the anti-apoptotic protein Bcl-2, thereby activating pro-apoptotic pathways. These combined effects substantially decrease tumor cell radioresistance [30].

### 2.4. Cell Invasion and Metastasis

Ionizing radiation can alter tumor cell phenotypes or the tumor microenvironment, increasing the invasiveness of residual tumor cells and thereby raising the likelihood of metastasis [31]. In radioresistant prostate cancer models, aberrant activation of the PI3K/AKT/mTOR signaling pathway significantly promotes EMT and the formation of cancer stem cell (CSC) phenotypes. This is evident from the downregulation of the epithelial marker E-cadherin and the upregulation of mesenchymal markers such as N-cadherin, Vimentin, OCT3/4, SOX2, and α-smooth muscle actin (SMA). These changes markedly enhance the invasive capability and sphere-forming ability of tumor cells while increasing their resistance to radiotherapy [32]. In another prostate cancer study, the overexpression of the epithelial cell adhesion molecule (EpCAM) significantly elevated the phosphorylation levels of key PI3K/AKT/mTOR pathway molecules, including p-AKT, p-mTOR, and p-ribosomal protein S6 kinase (S6K), further activating this pathway. High EpCAM expression also promoted the transition of cells from an epithelial state to a mesenchymal state by downregulating E-cadherin and upregulating Vimentin and N-cadherin, thereby enhancing cell migration and invasion [33]. In gastric cancer models, the high expression of caldesmon 1 (CALD1) further underscores the pivotal role of the PI3K/AKT/mTOR pathway in EMT induction. CALD1 upregulates PI3K, p-AKT, and p-mTOR expression while inhibiting phosphatase and tensin homolog (PTEN) expression, significantly activating this pathway. This activation leads to the downregulation of E-cadherin and Claudin-1 and the upregulation of N-cadherin and Vimentin, enhancing tumor cell migration and invasion. Additionally, PI3K/AKT inhibitors can partially reverse the EMT phenotypic changes induced by CALD1, suggesting that CALD1 regulates EMT via the PI3K/AKT/mTOR pathway [34].

### 2.5. Autophagy and Hypoxia

The hypoxic state of solid tumors is a critical factor contributing to the suboptimal efficacy of radiotherapy. A combination of physical, chemical, and biological processes reduces the sensitivity of hypoxic tumor cells to ionizing radiation and induces hypoxia-related therapeutic resistance [35].

Hypoxia-inducible factor 1-alpha (HIF-1α) is a key transcription factor that supports tumor growth, metabolism, invasion, and metastasis under hypoxic microenvironments [36]. Radiotherapy activates the PI3K/AKT/mTOR signaling pathway, promoting HIF-1α protein synthesis and enhancing its interaction with heat shock protein 90 (Hsp90), thereby stabilizing HIF-1α. The inhibition of Hsp90 or the PI3K/AKT/mTOR pathway significantly reduces radiotherapy-induced HIF-1α expression and diminishes vascular endothelial growth factor (VEGF) levels, weakening angiogenesis and decreasing the radioresistance of lung cancer cells. Additionally, HIF-1α collaborates with glucose transporter 1 (Glut-1) to drive hypoxia-induced radioresistance. HIF-1α regulates Glut-1 expression via the PI3K/AKT/mTOR pathway, facilitating glucose uptake and glycolytic metabolism in tumor cells to provide energy support. The knockdown of HIF-1α or Glut-1 genes or the inhibition of the PI3K/AKT/mTOR pathway reduces hypoxia-induced radioresistance, further validating this mechanism [37].

Under mild hypoxia, autophagy serves as one of the main drivers of tumor cell radioresistance [37]. Studies have shown that cellular Jun proto-oncogene (c-Jun) knockdown suppresses autophagy activation by inhibiting the PI3K/AKT/mTOR signaling pathway, thereby increasing radiation sensitivity. This is evidenced by the upregulated expression of p-PI3K, p-AKT, p-mTOR, and Sequestosome-1 (P62), alongside the downregulated expression of the autophagy-related molecule Microtubule-Associated Protein 1 Light Chain 3 II (LC3-II) [38]. Furthermore, high expression of neural precursor cell expressed, developmentally downregulated 8 (NEDD8) enhances autophagy activation by inhibiting the PI3K/AKT/mTOR pathway, significantly increasing tumor cell radioresistance. Specifically, NEDD8 overexpression elevates protein levels of autophagy-related molecules Beclin-1, Autophagy-related gene 5 (ATG5), and LC3-II, whereas NEDD8 knockdown reduces these molecules’ expression and markedly decreases tumor cell radioresistance [39]. In hepatocellular carcinoma models, the inhibition of mTOR phosphorylation significantly activates autophagy and enhances radiation-induced apoptosis in tumor cells. Under combined radiotherapy conditions, the suppression of mTOR phosphorylation increases autophagosome formation, upregulates the expression of autophagy markers LC3-II and Beclin-1, and downregulates P62 expression, indicating significantly enhanced autophagic activity [40].

### 2.6. Cancer Stem Cells

Cancer stem cells (CSCs) are a subset of tumor cells with self-renewal capacity and tumor-initiating potential, capable of generating a large population of non-tumorigenic progeny. Because CSCs can initiate new tumors, their incomplete elimination during anticancer treatment may lead to tumor recurrence and metastasis.

The PI3K/AKT/mTOR signaling pathway plays a critical role in CSCs by maintaining stemness, regulating proliferation and differentiation, promoting EMT, facilitating migration, and modulating autophagy [41]. This pathway stabilizes SOX2 within the nucleus by preventing its proteasomal degradation and cytoplasmic retention, thereby maintaining normal stem cell characteristics and functionality [42]. Studies have demonstrated that radiation activates the PI3K/AKT pathway, upregulating SOX2 expression and enhancing the self-renewal and differentiation capacity of colorectal CSC-like cells. Furthermore, the inhibition of the PI3K/AKT pathway reduces the expression of SOX2 and CSC markers, such as Cluster of Differentiation 44 (CD44), and impairs the migration and invasion capabilities of CSCs [43]. In breast cancer CSC models, HIF-2α upregulates CD44 expression via the PI3K/AKT/mTOR pathway, significantly enhancing CSC survival and radioresistance [44]. Similarly, in radioresistant prostate cancer cell models, activation of the PI3K/AKT/mTOR pathway promotes EMT and reinforces CSC phenotypes. This phenotypic change is characterized by the downregulation of E-cadherin and the upregulation of mesenchymal markers such as Vimentin and N-cadherin, leading to a marked increase in cell migratory capacity [32].

## 3. The Impact of Different PI3K/AKT/mTOR Isoforms on Radioresistance

### 3.1. PI3K Isoforms

PI3K is divided into three classes: I, II, and III, each consisting of specific regulatory and catalytic subunits with distinct substrate specificities and functions. These classes regulate cellular signaling and metabolic pathways in diverse ways [45]. Among them, Class I PI3Ks (PI3Kα, PI3Kβ, PI3Kγ, and PI3Kδ) are the focus of cancer research and the primary subject of this review [46].

PI3Kα regulates key cellular processes such as survival and proliferation, and its hyperactivation is a major contributor to cancer therapy resistance [47]. Danyaei et al. demonstrated that CRISPR/Cas9-mediated knockout of the PI3Kα gene in MDA-MB-231 breast cancer cells, combined with radiotherapy, significantly enhanced apoptosis, inhibited proliferation, and reduced angiogenesis within the tumor microenvironment [48]. In esophageal squamous cell carcinoma (ESCC) models, the PI3Kα-selective inhibitor CYH33 combined with radiotherapy suppressed PI3K/AKT signaling, reduced the phosphorylation of p-AKT and Forkhead Box O1 (FOXO1), delayed DSB repair, induced G2/M cell cycle arrest, and enhanced apoptosis while improving the tumor immune microenvironment [49]. Additionally, Korovina et al. found that the PI3Kα inhibitor Alpelisib demonstrated significant radiosensitizing effects in head and neck squamous cell carcinoma (HNSCC) models. However, resistance was observed in some models, which could be overcome by co-inhibiting β1 integrin, further enhancing radiotherapy efficacy [50].

PI3Kβ plays a crucial role in PTEN-deficient tumors and is associated with thrombosis, male fertility, and fragile X syndrome [51]. Regarding DNA damage repair, studies have shown that PI3Kβ recruits the Nijmegen Breakage Syndrome 1 (Nbs1) protein to activate the MRE11-RAD50-NBS1 complex (MRN) complex and ATM/ATR signaling pathways, significantly enhancing DSB repair capacity [52]. Furthermore, PI3Kβ interacts with Replication Factor C Subunit 1 (RFC1) to regulate RFC complex assembly and promotes DNA replication and repair through direct interaction with Proliferating Cell Nuclear Antigen (PCNA) [53,54].

PI3Kγ is expressed in most human tissues, predominantly in tumor-associated macrophages (TAMs), where it is abnormally activated and maintains the immune-suppressive M2 polarization phenotype, thereby weakening anti-tumor immune responses [55]. The inhibition of PI3Kγ can reprogram TAMs into pro-inflammatory M1 phenotypes, enhancing CD8+ T cell-mediated anti-tumor immunity and significantly improving radiotherapy efficacy [56]. Additionally, the PI3Kδ/γ dual inhibitor BR101801 combined with radiotherapy reduces the proportion of regulatory T cells (Tregs) in the tumor microenvironment, enhances CD8+ T cell activity, and significantly increases the occurrence of the abscopal effect [57].

**Figure 2:**
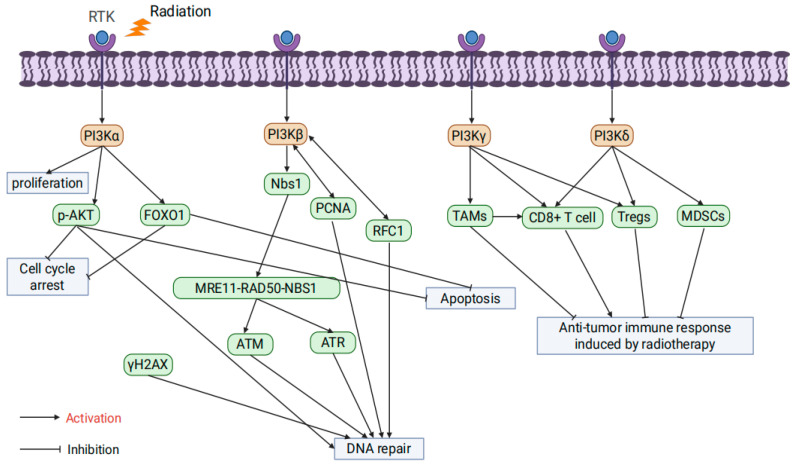
The influence of PI3K isoform specificity on tumor radioresistance.

PI3Kδ is predominantly expressed in hematopoietic cells but also plays a role in various tumor cells [58]. PI3Kδ inhibitors combined with radiotherapy can reverse the immunosuppressive state of the tumor microenvironment by reducing Treg and myeloid-derived suppressor cell (MDSC) infiltration, thereby enhancing CD8+ T cell activity [59,60]. Furthermore, PI3Kδ inhibitors such as Idelalisib, when used with radiotherapy, significantly enhance DNA damage response and promote apoptosis. However, in some models, they also increase radiotherapy-induced toxicity [61,62] (Figure 2).

### 3.2. AKT Isoforms

AKT consists of three isoforms: AKT1, AKT2, and AKT3, each playing unique roles in physiological functions and oncogenic mechanisms. AKT1 is essential for placental development, cell proliferation, and growth [63]; AKT2 is critical for maintaining glucose homeostasis [64]; and AKT3 is vital for brain development as its absence results in brain underdevelopment in mice [65]. Increasing evidence highlights the distinct roles of AKT1, AKT2, and AKT3 in tumor progression and radiation resistance [66].

AKT1 significantly affects radioresistance by regulating DNA repair pathways. Studies by Toulany et al. showed that AKT1 activation enhances the activity of DNA-PKcs, promoting NHEJ repair and increasing radiation resistance. Inhibiting AKT1 delays DSB repair and enhances apoptosis through cleaved PARP upregulation [67]. Further research revealed that AKT1 is crucial during both the initiation and completion phases of NHEJ repair, facilitating Ku70/Ku80 binding to DNA. AKT1 inhibition prolongs G2/M phase arrest, thereby enhancing radiotherapy-induced cytotoxicity [68,69]. HER3-dependent nuclear AKT1 activation further promotes NHEJ repair by enhancing DNA-PKcs activity, while HER3 inhibitors reduce AKT1 activity and decrease radioresistance [70]. Other studies demonstrated that limiting AKT1 phosphorylation delays DSB repair and significantly increases radiation-induced cytotoxicity [71]. In HR, AKT1 enhances repair efficiency by promoting Rad51 focus formation and colocalization with γ-H2AX. Knocking down AKT1 leads to the accumulation of unrepaired DSBs, thereby decreasing radioresistance [72]. Additionally, activating mutations of AKT1 (e.g., TDSD and E17K) accelerate γ-H2AX focus resolution, improve DSB repair, and enhance radiation resistance [73].

Research by Seiwert et al. revealed that AKT2 regulates radioresistance by suppressing autophagy. Radiation-induced DSBs typically activate autophagy as a protective mechanism, but AKT2 suppresses autophagy initiation via mTOR signaling, thereby improving radioresistance [74]. In HR, AKT2 promotes Rad51 focus formation and accelerates radiation-induced DNA repair; conversely, AKT2 knockdown reduces Rad51 recruitment, leading to DNA damage accumulation and increased radiation-induced cell death [75]. AKT2 also interacts with DNA-PKcs and MRE11, participating in alternative NHEJ repair pathways, suggesting its involvement in multiple DNA repair mechanisms [76]. However, studies indicated that in K-RAS mutant cells, AKT1 and AKT3 form stable complexes with DNA-PKcs, while AKT2 does not, suggesting that AKT2’s role in radioresistance regulation may be less critical in specific contexts [69].

**Figure 3:**
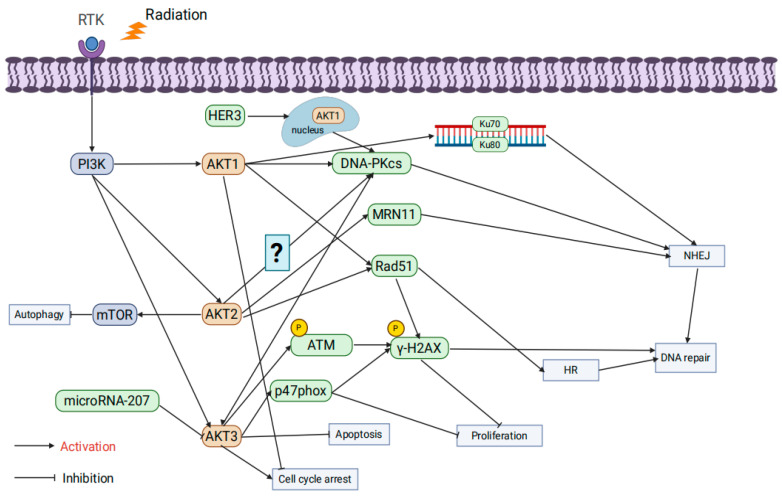
The influence of AKT isoform specificity on tumor radioresistance.

AKT3 exhibits multifaceted roles in regulating radiation resistance. Research has shown that microRNA-207 directly targets and suppresses AKT3 expression, inhibiting anti-apoptotic signaling and enhancing radiation-induced apoptosis [77]. Moreover, AKT3 phosphorylates the NADPH oxidase subunit p47phox, significantly increasing reactive oxygen species (ROS) levels, which triggers DDR and inhibits tumor cell proliferation. However, in the presence of p53 mutations or deletions, this mechanism facilitates immune evasion and enhances tumor growth [78]. In colorectal cancer models, AKT3 regulates DNA repair through the IL-13/mTOR signaling pathway, showing differential effects in tumors and normal tissues: reduced AKT3 activation in tumors decreases cell survival, while elevated IL-13 levels in normal tissues enhance AKT3 and mTOR signaling, promoting tissue repair [79]. In glioblastoma models, AKT3 gene amplification significantly increases the expression of DNA repair proteins, as evidenced by a significant increase in p-γ-H2AX foci and strong phosphorylation of ATM following γ-irradiation in AKT3-overexpressing cells compared with the empty vector group. This activation confers strong resistance to radiotherapy and chemotherapy (e.g., temozolomide), suggesting that AKT3 amplification may be a key mechanism underlying radiation resistance [80] (Figure 3).

### 3.3. mTOR Isoforms

mTOR is an evolutionarily conserved serine/threonine protein kinase that forms two multiprotein complexes: mTORC1 and mTORC2. mTORC1 primarily regulates cell growth, while mTORC2 is involved in the regulation of cell survival and proliferation [81].

The mTORC1 complex comprises three core proteins: mTOR, mammalian lethal with SEC13 protein 8 (mLST8), and Regulatory-Associated Protein of mTOR (RAPTOR) [82]. Its abnormal activation is closely associated with radiation resistance. At the metabolic level, studies have shown that Fusobacterium nucleatum promotes radiation resistance in nasopharyngeal carcinoma through the Solute Carrier Family 7 Member A5 (SLC7A5)/leucine-mTORC1 pathway. Leucine uptake activates mTORC1 signaling, inhibiting radiation-induced oxidative stress and apoptosis. However, a leucine-restricted diet suppresses mTORC1 activity, significantly enhancing the efficacy of radiotherapy, underscoring the critical role of mTORC1 in amino acid metabolism regulation [83]. Additionally, negative regulators of mTORC1, such as DEP domain-containing mTOR-interacting protein (DEPTOR), also play important roles in radiation resistance. Radiation induces DEPTOR degradation, activating mTORC1 signaling to inhibit apoptosis and enhance tumor cell survival. Stabilizing DEPTOR significantly inhibits mTORC1 signaling, increasing the sensitivity of hypopharyngeal cancer cells to radiation therapy [84]. In the context of DNA damage repair, mTORC1 directly regulates HR and NHEJ pathways through its downstream molecules S6K1 and Eukaryotic translation initiation factor 4E-binding protein 1 (4E-BP1), enhancing the efficiency of DNA repair and reducing cell death caused by radiation [85]. However, inhibiting mTORC1 alone may trigger compensatory activation of Akt signaling, thereby diminishing the radiosensitization effect. When mTORC1 inhibitors (e.g., rapamycin) are combined with Akt inhibitors (e.g., MK2206), DSB repair is significantly suppressed, further enhancing the efficacy of radiation therapy. This finding highlights the potential of combination targeting strategies [86]. Interestingly, the function of mTORC1 is significantly influenced by the metabolic environment. Murata et al. reported that under nutrient-deficient conditions (e.g., glucose restriction), the abnormal activation of mTORC1 decreases the radioresistance of hepatocellular carcinoma cells. This may be associated with a metabolic stress-induced energy crisis, further indicating that the role of mTORC1 in radiation resistance varies depending on the tumor microenvironment [87].

**Figure 4:**
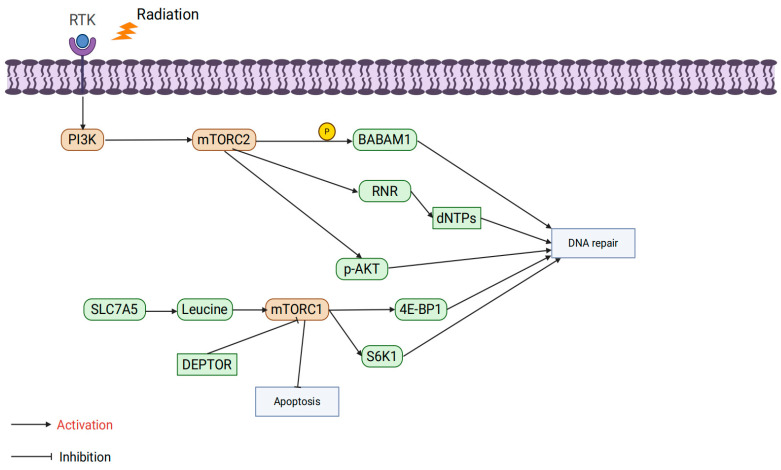
The influence of mTOR isoform specificity on tumor radioresistance.

The mTORC2 complex consists of rictor, mTOR, PPR5, G-protein β-subunit-like protein (GβL), DEPTOR, and stress-activated protein kinase-interacting protein 1 (SIN1) [88]. Although studies on mTORC2’s role in radioresistance are limited, existing data highlight its critical role in DNA damage repair. Research by Kalpongnukul et al. demonstrated that mTORC2 enhances the activity of DNA repair pathways by phosphorylating BABAM1 (BRISC and BRCA1-A complex member 1), thereby increasing radiation resistance in glioblastoma [89]. Additionally, Tian et al. found that mTORC2 phosphorylates ribonucleotide reductase (RNR), promoting deoxynucleotide synthesis and accelerating DNA replication. This sustained DNA replication capacity plays a crucial role in both gemcitabine resistance and radiation resistance [90]. Currently, specific inhibitors targeting mTORC2 alone are scarce, and most studies focus on dual mTORC1/2 inhibitors. For example, suppressing the phosphorylation of mTORC2 downstream target p-Akt has been shown to significantly enhance the efficacy of radiation therapy [91,92,93]. These findings suggest that mTORC2 plays an equally important role in regulating DNA repair and metabolism, and the dual targeting of mTORC1 and mTORC2 may be an effective strategy for overcoming radiation resistance in the future (Figure 4).

## 4. Preclinical Studies of PI3K/AKT/mTOR Inhibitors Combined with Radiotherapy

### 4.1. Digestive System Tumors

Digestive system tumors are among the most prevalent malignancies globally, with treatment strategies typically involving surgery, radiotherapy, and systemic chemotherapy [94]. However, the heterogeneity and radiation resistance of these tumors significantly limit the efficacy of radiotherapy.

In colorectal cancer (CRC) models, the combination of the mTOR inhibitor temsirolimus and the autophagy inhibitor chloroquine significantly enhanced the antitumor effects of radiotherapy, as evidenced by a marked increase in apoptosis rates. This combination therapy inhibited the phosphorylation of key mTOR signaling proteins, p-S6 and p-4E-BP1, and reduced levels of LC3-II and p62, thereby suppressing autophagy. In vivo experiments demonstrated that this combination therapy effectively inhibited the growth of CRC xenograft tumors without notable toxicity [95]. Another study showed that the dual PI3K/mTOR inhibitor BEZ235 combined with radiotherapy delayed DNA damage repair and significantly reduced the expression of angiogenesis-related factors VEGF-A and HIF-1α. In vivo, the combination therapy substantially inhibited CRC tumor growth, with the maintenance therapy group showing particularly pronounced effects [96].

In hepatocellular carcinoma models, the dual PI3K/mTOR inhibitor PKI-587 and the natural compound Tenacissoside H (TEH) demonstrated radiosensitization potential. PKI-587 interfered with NHEJ and HR repair pathways, delaying the dissipation of γ-H2AX foci induced by radiation and enhancing radiation-induced apoptosis. In vivo, the combination therapy significantly reduced tumor volumes without causing notable toxicity [19]. TEH, on the other hand, enhanced radiotherapy efficacy by downregulating PI3K/Akt/mTOR signaling activity and inducing autophagy. It specifically increased the LC3-II/LC3-I ratio, Beclin-1, and ATG5 expression, while reducing the anti-apoptotic protein Bcl-2 and upregulating the pro-apoptotic protein Bax. The combination of TEH and radiotherapy significantly reduced tumor cell viability and showed enhanced tumor inhibition in in vivo models [97].

The dual PI3K/mTOR inhibitor PF-04691502 also exhibited radiosensitizing effects in gastroenteropancreatic neuroendocrine tumor models. The study compared three administration schedules: simultaneous, pre-radiotherapy, and delayed post-radiotherapy. The group receiving PF-04691502 48 h after radiotherapy showed the most significant tumor volume reduction without causing notable toxicity to normal tissues [98]. In pancreatic cancer models, the mTOR inhibitor INK128 combined with radiotherapy significantly delayed DNA damage repair and enhanced the cytotoxic effects of radiotherapy by disrupting the formation of the Eukaryotic initiation factor 4F (eIF4F) cap complex, thereby inhibiting protein translation. The radiosensitization effect was most pronounced when INK128 was administered either 1 h before or within 6 h after radiotherapy. Delaying administration beyond 6 h post-radiotherapy markedly reduced its radiosensitization effects, emphasizing the critical role of drug-radiotherapy timing in combination treatments [99].

### 4.2. Genitourinary System Tumors

Radiotherapy is a crucial therapeutic option for prostate cancer, demonstrating significant efficacy across all stages of the disease [100]. Studies have shown that the dual PI3K/mTOR inhibitor NVP-BEZ235 significantly reduces the survival rate of prostate cancer PC-3 cells post-radiation. Combined treatment not only induces G2/M cell cycle arrest but also markedly enhances apoptosis rates and delays DNA damage repair [101].

In renal cell carcinoma (RCC) models, the combination of the mTOR inhibitor Everolimus and the Survivin inhibitor YM155 (EY-L) significantly potentiates radiosensitization. In vitro experiments demonstrated that EY-L reduces the clonogenic capacity of RCC cells, prolongs the persistence of the DNA damage marker γ-H2AX, and suppresses DNA repair by inhibiting the expression of p-p70S6K and Poly(ADP-ribose)polymerase 1 (PARP1). In vivo studies further revealed that EY-L combined with radiotherapy significantly inhibits tumor growth and increases CD8+ T cell infiltration within tumors, thereby amplifying antitumor immune responses [102].

In cervical cancer models, the combination of the PI3K inhibitor Alpelisib and the Aurora kinase inhibitor Alisertib exhibits notable radiosensitizing effects. In vitro data showed that Alpelisib enhances the cytotoxicity of Alisertib, reducing the IC50 of HeLa cells from 19,550 nM to 30.3 nM. Combined treatment significantly prolongs cell division time, induces mitotic failure, and leads to apoptosis or necrosis in 97.9% of cells. Molecular analyses revealed increased levels of cleaved PARP and caspase expression following combination therapy. In vivo experiments further validated that Alpelisib and Alisertib combination therapy significantly suppresses tumor growth in HeLa xenograft mouse models [103].

In bladder cancer models, the mTOR inhibitor Everolimus combined with radiotherapy exhibits significant radiosensitizing effects. In vitro studies revealed that Everolimus (RAD001) markedly reduces the clonogenic survival of bladder cancer cells and enhances radiotherapy efficacy by inhibiting downstream mTOR signaling, such as p-S6 phosphorylation, and regulating the cell cycle. The combination induces dual-phase cell cycle arrest at G0/G1 and G2/M phases, reduces the proportion of S-phase cells, downregulates cyclin D1, and upregulates p21 and p27 expression. In vivo experiments demonstrated that Everolimus combined with radiotherapy significantly reduces tumor weight in bladder cancer xenograft models by approximately 72% and increases p21 expression [104].

### 4.3. Respiratory System Tumors

Radiotherapy is a key treatment modality for lung cancer, with approximately 77% of patients being eligible for this approach. However, radiotherapy remains underutilized in clinical practice [105]. In small-cell lung cancer (SCLC) models, the combination of PI3K/mTOR inhibitors BEZ235 and GSK2126458 with radiotherapy significantly reduced the survival rates of SCLC cells, increased apoptosis rates to 24–32%, and markedly suppressed clonogenic capacity. The combination therapy enhanced DNA damage, evidenced by the significant upregulation of γ-H2AX and p-ATM expression levels [106]. In NSCLC, the combination of the MEK inhibitor Trametinib, the mTOR inhibitor Temsirolimus, and radiotherapy demonstrated synergistic effects. The combined treatment reduced the survival rates of A549 and NCI-H460 cells under 8 Gy irradiation by approximately 50%, significantly prolonged G2/M phase cell cycle arrest, increased γ-H2AX expression, and elevated radiation-induced apoptosis. In A549 xenograft nude mouse models, tumor growth inhibition in the combination therapy group outperformed both radiotherapy and single-drug treatments [107]. Furthermore, Seol et al. compared the effects of different PI3K inhibitors combined with radiotherapy, finding that both the PI3K-α inhibitor GDC0032 and the pan-PI3K inhibitor GDC0941 reduced A549 cell survival rates to 25%, exhibiting similar radiosensitizing effects. However, GDC0032 demonstrated lower toxicity [108].

Radiotherapy is also a crucial treatment for nasopharyngeal carcinoma (NPC) and certain HNSCC in patients who are inoperable [109]. In NPC models, the mTOR inhibitor temsirolimus dose-dependently induced caspase-dependent apoptosis and significantly decreased the radioresistance of the resistant cell line C666-1-r. In vivo experiments demonstrated that temsirolimus delayed tumor growth in C666-1-r models, reducing tumor formation rates from 80% to 20–55% without notable toxicity [110]. In HNSCC models, the DNA-PK inhibitor AZD7648 combined with radiotherapy significantly delayed the repair of DSBs, induced G2/M phase cell cycle arrest, and suppressed cell proliferation and clonogenic capacity. Additionally, the mTOR inhibitor Sapanisertib exhibited radiosensitizing effects in HPV+ cell lines but showed limited efficacy in HPV− cell lines. In contrast, the dual-function inhibitor CC-115, which targets both DNA-PK and mTOR, significantly enhanced radiotherapy outcomes in both HPV+ and HPV− cell lines, suggesting broader clinical utility [111]. However, a study by Glorieux et al. indicated variability in the radiosensitizing effects of PI3K inhibitors in HNSCC cells. While BKM120 and GDC0980 effectively inhibited p-Akt and p-mTOR expression and weakened DNA repair in some cell lines, their overall radiosensitizing efficacy fell short of expectations. These findings highlight the need for patient-specific selection strategies when employing PI3K inhibitors in clinical applications [112].

### 4.4. Breast Cancer

Radiotherapy is a critical component of multimodal breast cancer management, widely applied in early-stage, locally advanced, and metastatic cases [113]. In triple-negative breast cancer (TNBC) models with a high risk of brain metastasis, the AKT inhibitor Ipatasertib significantly reduced the survival rates of MDA-MB-231BR cells. However, Ipatasertib did not affect cell migration, whereas the knockout of the AKT1 isoform unexpectedly increased migration potential [114]. Johnson et al. investigated the radiosensitizing effects of various AKT inhibitors and found that MK-2206 reduced AKT1 expression and induced PARP cleavage in MDA-MB-231 cells after 24 h of treatment. When combined with 4 Gy radiation, MK-2206 significantly enhanced radiation-induced PARP cleavage. In contrast, perifosine reduced AKT1 expression but did not show significant synergistic apoptotic effects, while A-674563 and AZD5363 neither reduced AKT1 levels nor induced apoptosis [115].

Dual PI3K/mTOR inhibitors have shown remarkable radiosensitizing effects across different breast cancer subtypes. Gasimli et al. demonstrated that PKI-402 exhibited strong radiosensitizing effects in Luminal A subtype (MCF-7) and breast CSCs. In MDA-MB-231 cells, PKI-402 combined with radiotherapy significantly increased DSB levels, but this effect was not observed in MCF-7 or breast CSCs. Mechanistically, PKI-402 reduced phosphorylated Glycogen synthase kinase 3 beta (GSK-3β) and Proline-rich AKT substrate of 40 kDa (PRAS40) levels, thereby modulating mTORC1-related signaling pathways. In MCF-7 cells, PKI-402 further activated apoptotic signals by increasing the phosphorylated Bcl-2-associated agonist of cell death (BAD) [116]. Additionally, Masoumi et al. investigated the combined effects of the PI3K/mTOR inhibitor NVP-BEZ235 and the SIRT1 activator SRT1720 under radiotherapy. The study revealed that both NVP-BEZ235 and SRT1720 alone significantly reduced the survival rates of MCF-7 cells, with combined treatment yielding even greater effects. In IL-6 pretreated cells, the combination reduced survival rates to 43% of the control group. Furthermore, combined therapy significantly increased apoptosis and necrosis rates, thereby enhancing the radiosensitizing effect of the treatment [117].

### 4.5. Central Nervous System Tumors

Although radiotherapy is a key treatment modality for GBM, its efficacy remains limited [118]. Studies have shown that the PI3K-α inhibitor GDC0032, in combination with radiotherapy, significantly reduced the survival rates of LN229 and GL261-luc cells to 27% and 19%, respectively. Combined treatment notably prolonged the persistence of γ-H2AX foci, indicating inhibited DNA damage repair. Furthermore, GDC0032 significantly reduced p-AKT levels, achieving similar effects to the pan-PI3K inhibitor GDC0941 but with lower toxicity. In vivo experiments demonstrated that GDC0032 combined with radiotherapy significantly slowed tumor growth in GL261-luc mouse models and markedly improved survival times [119].

The radiosensitizing effects of the PI3K/mTOR inhibitor PI-103 vary depending on the molecular context. In DNA-PK-proficient MO59K cells, PI-103 combined with radiotherapy reduced the clonogenic survival fraction from 0.63 to 0.41, delayed DNA damage repair, and extended the persistence of γ-H2AX and p53-binding protein 1 (53BP1) foci. Additionally, PI-103 significantly promoted apoptosis by inducing cleaved PARP and cleaved caspase-3. Conversely, in DNA-PK-deficient MO59J cells, PI-103 unexpectedly accelerated DNA repair, marked by LC3B-II upregulation and p62 downregulation, inducing protective autophagy and increasing the survival fraction from 0.16 to 0.23 [120]. The AKT inhibitor MK-2206 also exhibited differential effects across GBM cell lines. It showed no significant radiosensitizing effects in DK-MG cells but paradoxically increased radioresistance in SNB19 cells. Further investigations revealed that while MK-2206 significantly suppressed p-AKT levels, it induced compensatory activation of the mTOR pathway in SNB19 cells, elevating p-mTOR and p-S6 levels and thereby diminishing radiotherapy efficacy. However, when PI-103 was combined with MK-2206, mTOR pathway activity was effectively suppressed, autophagy was enhanced, and DNA repair was delayed, reversing the resistance caused by compensatory activation [121]. These preclinical studies show the broad application prospects of PI3K/AKT/mTOR inhibitors in cancer (Table 1).

## 5. Clinical Studies on PI3K/AKT/mTOR Inhibitors Combined with Radiotherapy

PI3K inhibitors have emerged as an important strategy in cancer-targeted therapy. These inhibitors, targeting different PI3K isoforms (PI3Kα, PI3Kβ, PI3Kγ, and PI3Kδ) through selective or pan-inhibition, have shown significant potential in clinical studies. In contrast, most AKT inhibitors are pan-inhibitors, targeting AKT1, AKT2, and AKT3 through ATP-competitive or allosteric mechanisms. However, this broad-spectrum inhibition often results in higher adverse effects, limiting their clinical application. Despite these challenges, the approval of the AKT inhibitor Capivasertib in 2023 for HR+/HER2- advanced metastatic breast cancer marked a significant breakthrough in the clinical translation of this drug class [122]. As the downstream component of the PI3K/AKT/mTOR pathway, mTOR inhibitors mainly target the mTORC1 complex to suppress cell proliferation and metabolism and are widely applied in the treatment of solid and hematologic tumors. Currently, multiple PI3K/AKT/mTOR inhibitors are undergoing clinical trials (Table 2).

Alpelisib, a selective PI3Kα inhibitor, has garnered attention for its radiosensitization effects, particularly in HNSCC. In a phase I trial, Alpelisib combined with cisplatin and concurrent chemoradiotherapy demonstrated good tolerability and achieved local tumor control in most patients with advanced HNSCC. However, some patients experienced distant metastasis, necessitating further phase II trials to evaluate its clinical efficacy [123]. Another study reported that Alpelisib combined with cetuximab and radiotherapy was well-tolerated, though some patients developed severe mucositis and leukopenia, indicating a need for optimized toxicity management [124]. Buparlisib, a pan-PI3K inhibitor, significantly reduced hypoxia in tumor regions when combined with low-dose thoracic radiotherapy (20 Gy) in NSCLC patients during a phase I trial. This reduction was closely associated with decreased radioresistance and good tolerability [125]. However, dose-limiting toxicity has restricted its further development. In another phase I trial involving newly diagnosed GBM patients, Buparlisib combined with temozolomide and radiotherapy demonstrated good biomarker suppression but was associated with high rates of hyperglycemia and elevated liver enzymes, limiting dose escalation and failing to establish a maximum tolerated dose (MTD) [126]. Voxtalisib, a dual PI3K/mTOR inhibitor, showed limited radiosensitization effects in high-grade glioma patients, with only a 4% partial response rate in clinical studies. Nevertheless, its safety profile was favorable [127].

Clinical studies on AKT inhibitors are relatively limited. Perifosine, a novel AKT inhibitor [128], was evaluated in a phase I trial combining daily oral doses (50–200 mg) with fractionated radiotherapy in various advanced solid tumors, including NSCLC, bladder cancer, prostate cancer, and esophageal cancer. It showed radiosensitization effects across multiple tumor types, with an infield response rate of 52% (11/21, including five partial responses and six complete responses) after a median follow-up of 10 months, with an MTD of 150 mg/day [129]. Nelfinavir, initially developed as an HIV protease inhibitor, was later found to downregulate phosphorylated AKT levels, demonstrating potential as a radiosensitizer [130,131]. In a phase I study, Nelfinavir combined with cisplatin and concurrent chemoradiotherapy achieved a remarkable 91% disease-free survival rate (RFS) at a median follow-up of 50 months in cervical cancer patients treated with 1250 mg/day. This regimen was well-tolerated [132]. In another study involving 11 pancreatic cancer patients, Nelfinavir combined with SBRT (40 Gy/5 fractions) showed promising feasibility and safety. Among the 10 patients who received radiotherapy, four underwent surgical resection, and all resected specimens exhibited negative margins. The median overall survival was 13 months despite the premature termination of the study [133]. However, a phase III trial in locally advanced pancreatic cancer failed to demonstrate significant improvements in progression-free survival (PFS) or overall survival (OS), highlighting the need for optimization in specific cancer types [134].

Temsirolimus, an mTOR inhibitor, demonstrated partial responses in patients with locally advanced NSCLC when combined with thoracic radiotherapy in a phase I trial. However, pulmonary toxicity, including pneumonia and hemorrhage, necessitated cautious dose adjustments, with an MTD of 15 mg weekly via intravenous administration [135]. In a phase II trial for GBM, temsirolimus combined with radiotherapy did not significantly improve the 12-month overall survival rate. Nonetheless, patients with high mTOR phosphorylation levels showed better outcomes, suggesting that specific molecular subgroups may benefit from mTOR inhibitors as radiosensitizers [136]. Everolimus, another mTOR inhibitor, achieved a 23% pathological complete response rate and a 2-year PFS of 50% when combined with carboplatin and radiotherapy in a phase I study for locally advanced esophageal cancer [137]. In locally advanced cervical cancer, Everolimus demonstrated good tolerability with dose escalation up to 10 mg/day, without irreversible toxicity [138]. However, in newly diagnosed GBM, 10 mg/day of Everolimus combined with temozolomide and radiotherapy failed to significantly improve PFS and was associated with high toxicity [139]. In a study involving patients with biochemical recurrence of prostate cancer after surgery, Everolimus combined with radiotherapy at escalating doses (5, 7.5, and 10 mg/day) resulted in 22% of patients experiencing grade 3 acute toxicity [140].

These studies collectively underscore the potential of PI3K/AKT/mTOR inhibitors as radiosensitizers in various solid tumors. Large-scale, randomized, controlled trials are essential to validate their efficacy and safety in clinical settings.

## 6. Future Research Directions

### 6.1. Molecular Design and the Targeted Optimization of Novel Inhibitors

Despite the significant potential of PI3K/AKT/mTOR pathway inhibitors in radiosensitization research, their clinical applications are still limited by systemic toxicity and insufficient target specificity. For instance, PI3K/AKT inhibitors frequently induce adverse reactions such as hypertension, hyperglycemia, and severe pneumonia, posing significant obstacles to their clinical use [141]. A large meta-analysis involving 6710 patients demonstrated that PI3K/AKT inhibitors are effective in patients with specific genetic mutations, but their safety profiles are relatively poor. Among these, Capivasertib has shown good efficacy and safety in solid tumors like breast cancer, while Idelalisib has exhibited remarkable effects in hematological malignancies such as chronic lymphocytic leukemia. These studies also revealed that AKT inhibitors are generally safer than PI3K inhibitors, which are more prone to causing pneumonia, though the underlying mechanism remains unclear [142].

Balancing efficacy and toxicity control remains a core challenge in clinical research, further driving the development of personalized inhibitors targeting different subtypes of the PI3K/AKT/mTOR pathway. Examples include Alpelisib as a selective PI3Kα inhibitor, GSK2636771 targeting PI3Kβ, Idelalisib and Zandelisib targeting PI3Kδ, and Eganelisib focusing on PI3Kγ, all of which demonstrate strategies for refined targeting. The development of these inhibitors not only enhances efficacy but also significantly reduces damage to normal tissues [143,144,145,146,147]. Additionally, inhibitor development for mTOR complexes has also become more refined. For instance, Everolimus has been developed as an mTORC1-specific inhibitor, while JR-AB2-011 is an mTORC2-specific inhibitor, offering more options for precise pathway regulation [148,149]. Future studies should focus on further optimizing molecular designs, particularly developing specific inhibitors for the three AKT isoforms, to reduce systemic toxicity and improve targeting. This will help ensure therapeutic efficacy while minimizing damage to normal tissues.

### 6.2. Combination Therapy Strategies

Combination therapy is a critical approach to enhancing the radiosensitization potential of PI3K/AKT/mTOR inhibitors. Studies have shown that the PI3K/AKT/mTOR pathway is often excessively activated after chemotherapy, closely associated with chemotherapy resistance [150]. Combining PI3K/AKT/mTOR inhibitors with standard chemotherapy not only reduces chemotherapy resistance but has also demonstrated significant sensitization effects in preclinical studies on cervical cancer and liver cancer [151]. In radiochemotherapy strategies, chemotherapy enhances the efficacy of radiotherapy through mechanisms such as increasing DNA damage, inhibiting DNA repair, altering the cell cycle, reducing hypoxic cells, and suppressing tumor repopulation [152].

Moreover, combining PI3K/AKT/mTOR inhibitors with radiotherapy and immunotherapy holds great promise. For instance, in preclinical studies on cervical cancer, the mTOR inhibitor RAD001 enhanced radiotherapy-induced PD-L1 expression. When combined with a PD-1 inhibitor, this approach further increased the number and cytotoxicity of CD8+ T cells, demonstrating synergistic effects in activating antitumor immunity [153]. Future research should explore the combination of PI3K/AKT/mTOR inhibitors with other therapeutic modalities, such as DNA damage repair inhibitors and angiogenesis inhibitors, aiming to achieve more precise and effective radiosensitization across various tumor types.

### 6.3. Identification of Predictive Tumor Biomarkers

With advancements in modern radiotherapy techniques, treatment plans can be highly individualized to match patients’ anatomical structures. However, challenges remain in regulating tumor biological characteristics, particularly the lack of biomarkers capable of accurately predicting tumor responses to radiotherapy. Such biomarkers are essential for personalized treatment decisions, dose design, and protocol optimization [154].

Currently, biomarkers such as phosphoinositide-dependent protein kinase 1 (PDK1) and annexin A6 (ANXA6) have shown potential in studies on the radiosensitization of PI3K/AKT/mTOR pathway inhibitors. In the Hepatocellular Carcinoma (HCC) study by Bamodu et al., aberrant PDK1 overexpression typified poorly differentiated HCC cells, with PDK1 mRNA upregulated 2.17-fold in HCC tissues versus normal liver tissues (*p* = 8.95 × 10^−6^). PDK1 independently activates PI3K/AKT/mTOR signaling, evident by enhanced p-PI3K, p-AKT, and p-mTOR expression. Knocking down PDK1 sensitizes HCC cells to radiotherapy: 2 Gy irradiation combined with siPDK1 reduces Mahlavu cell viability from 42% to 3% and inhibits clonogenicity by 91%, while Huh7 cell clonogenicity is suppressed by 99.8%. In radioresistant Huh7-R cells, PDK1 upregulation correlates with 24.16-fold increased ALDH1 activity and 7.03-fold higher side population cell proportions, indicating its role in cancer stemness [155]. In Chen et al.’s NPC research, ANXA6 was overexpressed in radioresistant NPC cells/tissues, positively correlating with radioresistance. ANXA6 promotes autophagy by inhibiting PI3K/AKT/mTOR, as siANXA6 reduces the LC3II/I ratio and elevates p62, while the PI3K inhibitor CAL101 reverses this autophagy suppression. Functionally, siANXA6 sensitizes CNE1R cells to 2 Gy irradiation, and CAL101 combination enhances tumor cell killing via PI3K/AKT/mTOR inhibition. Gene chip analysis shows that ANXA6 overexpression independently predicts poor NPC prognosis, positioning it as a novel radiotherapy prognosis biomarker [156]. These studies reveal PDK1 and ANXA6 as key drivers of radioresistance via PI3K/AKT/mTOR-mediated autophagy and stemness, offering therapeutic targets for HCC and NPC, but their universality and predictive value across different tumor types require further validation. Moreover, existing research predominantly focuses on single biomarkers, lacking systematic studies that integrate multidimensional analyses, such as molecular expression profiles, phosphorylation states, and liquid biopsy data. Future research should prioritize the development of predictive models incorporating multiple biomarkers, integrating diverse biological datasets to enhance clinical applicability and stability. This approach could enable more precise radiosensitization strategies.

## 7. Conclusions

The PI3K/AKT/mTOR signaling pathway plays a central role in modulating tumor radioresistance by systematically influencing tumor responses to radiotherapy through mechanisms such as DNA damage repair, cell cycle regulation, and the control of cell proliferation and apoptosis. The distinct roles of different PI3K, AKT, and mTOR isoforms in regulating radioresistance offer promising directions for precision-targeted therapies. Although preclinical studies demonstrate that targeting specific nodes within this pathway can significantly enhance the effects of radiotherapy, challenges such as limited target selectivity, resistance development, and toxicity continue to hinder clinical applications. Future research should focus on optimizing inhibitor design, developing combination treatment strategies, and identifying predictive biomarkers of sensitivity, aiming to achieve more precise and effective radiosensitization protocols. In summary, the PI3K/AKT/mTOR pathway represents a critical target for understanding tumor radioresistance and developing novel therapeutic strategies. Advances in this field hold the potential to bring new hope to the treatment of cancer.

## Figures and Tables

**Table 1:** PI3K/AKT/mTOR pathway inhibitors combined with radiotherapy in preclinical trials.

Inhibitor	Target	Population	Experimental Model	Radiotherapy Dose	Summary Outcome
Alpelisib	PI3K	Cervical cancer	In vivo (patient-derived orthotopic cervical cancer xenograft model)	2 Gy × 15	Combination therapy significantly reduced tumor volume in tumor model.
GDC0032 (Taselisib)	PI3K	Non-small-cell lung cancer	In vitro (cell lines: A549, LLC1) In vivo (LLC1 xenograft model)	In vitro: 2, 4, 6 Gy In vivo: 5 Gy × 1	It could significantly reduce the cell cloning rate, decrease radioresistance, and delay tumor growth in tumor models.
	PI3K	Glioblastoma	In vitro (cell lines: LN229, GL261-luc) In vivo (GL261-luc intracranial xenograft model)	In vitro: 1, 2, 3, 4, 6 Gy In vivo: 2 Gy × 5	Combined radiotherapy significantly reduced the cloning rate of GBM cells and decreased radioresistance.
CAL101 (Idelalisib)	PI3K	Glioblastoma	In vitro (LN229, GL261-luc)	1, 2 Gy	Combined radiotherapy could decrease radioresistance.
IPI145 (Duvelisib)	PI3K	Glioblastoma	In vitro (LN229, GL261-luc)	1, 2 Gy	Combined radiotherapy was more effective than radiotherapy alone.
GDC0941 (Pictilisib)	PI3K	Glioblastoma	In vitro (LN229, GL261-luc)	1, 2, 3, 4, 6 Gy	Combined radiotherapy could reduce the cell survival fraction.
BKM120 (Buparlisib)	PI3K	Head and neck squamous cell carcinoma	In vitro (cell lines: SCC154, SCC104, SCC47 (HPV^+^); SQD9, SCC61, CAL27 (HPV^−^))	0, 2, 4, 6 Gy	Only weak radiosensitization was observed in the HPV^+^ cell line SCC154, and most cell lines had no obvious synergistic effect.
GSK2126458	PI3K/mTOR	Small-cell lung cancer	In vitro (cell lines: SBC2, H446, DMS53, H446RR) In vivo (mouse model and subcutaneous allografts)	In vitro: 2, 4, 6 Gy In vivo: 6 Gy × 4	Significantly decreased the radioresistance of SCLC cells, including inhibiting proliferation, increasing apoptosis, and reducing tumor volume and weight.
NVP-BEZ235	PI3K/mTOR	Colorectal cancer	In vitro (cell lines: HCT116, HT29, SW480) In vivo (HCT116 xenograft tumor model)	In vitro: 1, 2, 3, 5 Gy In vivo: 2 Gy × 3	Significantly inhibited colorectal cancer cell viability, increased cell apoptosis, and significantly reduced tumor volume in xenograft models.
	PI3K/mTOR	Prostate cancer	In vitro (cell line: PC-3)	2, 4, 6, 8, 10 Gy	Pretreatment significantly decreased the radioresistance of PC-3 cells in a dose-dependent manner.
	PI3K/mTOR	Breast cancer	In vitro (MCF-7 cells)	2 Gy	Reduced cell viability and decreased radioresistance.
PF-04691502	PI3K/mTOR	Gastroenteropancreatic neuroendocrine tumors	In vitro (cell lines: QGP-1, BON, NT-3)	2, 4, 8 Gy	Use after radiotherapy could significantly increase cell apoptosis and show anti-proliferative effects.
PI-103	PI3K/mTOR	Glioblastoma	In vitro (cell lines: MO59K, MO59J)	0, 2, 3, 5, 6, 8 Gy	Radiosensitization effect on MO59K cells.
PKI-402	PI3K/mTOR	Breast cancer	In vitro (cell lines: MCF-7, MDA-MB-231, BCSCs, MCF-10A)	0, 2, 4, 6, 8, 10 Gy	Inhibited the colony formation of MCF-7 and BCSC and increased the apoptosis of MCF-7.
PKI-587	PI3K/mTOR	Hepatocellular carcinoma	In vitro (cell line: SK-Hep1) In vivo (SK-Hep1 xenograft model)	In vitro: 0, 2, 4, 6, 8 Gy In vivo: 2 Gy × 4	Significantly decreased the radioresistance of liver cancer cells and significantly inhibited tumor growth in xenograft models.
GDC-0068 (Ipatasertib)	AKT	Triple-negative breast cancer	In vitro (cell line: MDA-MB-231BR; parental line: MDA-MB-231)	0, 2, 4, 6 Gy	Significantly reduced cell viability at high concentrations (20 μM) and decreased radioresistance at IC20 concentrations (6 μM).
MK-2206	AKT	Triple-negative breast cancer	In vitro (MDA-MB-231)	4 Gy	Administration of 10 μM MK-2206 48 h after radiotherapy reduced Akt1 expression and synergistically enhanced radiotherapy-induced apoptosis.
	AKT	Glioblastoma	In vitro (cell lines: DK-MG, SNB19)	0, 2, 3, 5, 7, 8 Gy	Increased the radioresistance of SNB19 cells.
Everolimus	mTOR	Renal cell carcinoma	In vitro (cell lines: 786-O, Renca) In vivo (subcutaneous and orthotopic Renca xenografts, 786-O xenografts)	In vitro: 2 Gy In vivo: 10 Gy × 2	Everolimus could enhance the sensitivity of renal cell carcinoma to radiation.
	mTOR	Bladder cancer	In vitro (cell lines: UM-UC3, UM-UC5, UM-UC6, KU7, 253J-BV, 253-JP) In vivo (KU7 and 253J-BV xenograft models)	In vitro: 0, 1, 2, 3, 4 Gy In vivo: 3 Gy × 3	The combination therapy significantly reduced the colony formation rate of bladder cancer cells, showing an additive effect.
INK128	mTOR	Pancreatic carcinoma	In vitro (cell lines: PSN1, Panc1, Miapaca-2) In vivo (PSN1 xenograft model)	In vitro: 0, 2, 4, 6, 8 Gy In vivo: 6 Gy, 2 Gy × 4	Decreased radioresistance of pancreatic cancer cells.
Temsirolimus	mTOR	Colorectal cancer	In vitro (cell lines): HT-29, SW480	0, 2, 4, 6 Gy	The combination of temsirolimus and chloroquine decreased the radioresistance of CRC cells by co-inhibiting mTOR and autophagy.
	mTOR	Non-small-cell lung cancer	In vitro (cell lines: A549, NCI-H460) In vivo (A549 xenograft model)	In vitro: 0, 2, 4, 6, 8 Gy In vivo: 2 Gy × 4	It reduced the colony formation rate of cancer cells and enhanced radiation-induced apoptosis, reducing tumor volume in in vivo models.
	mTOR	Nasopharyngeal carcinoma	In vitro (cell lines: 5-8F, HNE-1, C666-1, 6-10B, CNE-2; radio-resistant cell line: C666-1-r) In vivo (C666-1-r xenograft model)	In vitro: 0, 2.5, 5, 10, 20, 40 Gy In vivo: no mention	It could significantly decrease the radioresistance of nasopharyngeal carcinoma cells. In xenograft models, temsirolimus reduced the tumor formation rate in a dose-dependent manner.
Sapanisertib	mTOR	Head and neck squamous cell carcinoma	In vitro (cell lines: CAL33 (HPV^−^), UD-SCC-2 (HPV^+^), UM-SCC-47 (HPV^+^), HSC4 (HPV^−^); normal cells: SBLF7/SBLF9 fibroblasts, HaCaT keratinocytes)	2 Gy	Combination with radiotherapy increased cell death and G2/M arrest and reduced clonogenicity.

**Table 2:** PI3K/AKT/mTOR pathway inhibitors combined with radiotherapy in clinical trials.

Inhibitor	Target	Population	Phase	ClinicalTrials.Gov Identifier
Alpelisib	PI3K	Head and Neck Squamous Cell Cancer	1	NCT02282371 Start date: October 2014 Completion date: October 2021
	PI3K	Head and Neck Cancer	1	NCT02537223 Start date: September 2015 Completion date: February 2020
Buparlisib	PI3K	Carcinoma, Non-Small-Cell Lung	1	NCT02128724 Start date: April 2013 Completion date: October 2017
	PI3K	Head and Neck Cancer	1	NCT02113878 Start date: September 2014 Completion date: January 2022
	PI3K	Glioblastoma	1	NCT01473901 Start date: December 2011 Completion date: May 2017
Voxtalisib	PI3K/mTOR	Glioblastoma	1	NCT00704080 Start date: August 2008 Completion date: February 2013
Paxalisib	PI3K/mTOR	Brain Metastases	1	NCT04192981 Start date: December 2019 Completion date: December 2025
	PI3K/mTOR	Diffuse Midline Gliomas	2	NCT05009992 Start date: October 2021 Completion date: June 2029
	PI3K/mTOR	Glioblastoma	2/3	NCT03970447 Start date: July 2019 Completion date: June 2028
Capivasertib	AKT	Breast Cancer	2	NCT06607757 Start date: December 2024 Completion date: August 2026
Ipatasertib	AKT	Head and Neck Cancer	1	NCT05172245 Start date: September 2022 Completion date: June 2026
Nelfinavir	AKT	Glioblastoma	1	NCT00694837 Start date: March 2009 Completion date: January 2013
	AKT	Oligometastases	2	NCT01728779 Start date: January 2014 Completion date: December 2020
	AKT	Cervical Cancer	1	NCT01485731 Start date: January 2012 Completion date: February 2015
	AKT	Pancreatic Cancer	1	NCT01068327 Start date: November 2007 Completion date: February 2015
	AKT	Pancreatic Cancer	2	NCT01959672 Start date: September 2013 Completion date: December 2018
	AKT	Glioblastoma	1	NCT01020292 Start date: April 2009 Completion date: December 2017
	AKT	Non-Small-Cell Lung Cancer	1/2	NCT00589056 Start date: June 2007 Completion date: March 2012
	AKT	Pancreatic Cancer	1/2	NCT00589056 Start date: March 2016 Completion date: June 2021
	AKT	Cervical Cancer	1	NCT02363829 Start date: February 2015 Completion date: February 2020
	AKT	Head and Neck Squamous Cell Cancer	2	NCT02207439 Start date: July 2014 Completion date: May 2022
Everolimus	mTOR	High-Grade Glioma	2	NCT05843253 Start date: August 2024 Completion date: August 2034
	mTOR	Neuroendocrine Tumors	3	NCT05918302 Start date: October 2023 Completion date: July 2028
	mTOR	Diffuse Intrinsic Pontine Glioma	3	NCT05476939 Start date: September 2022 Completion date: September 2031
	mTOR	Glioblastoma	1/2	NCT00553150 Start date: March 2009 Completion date: November 2019
	mTOR	Head and Neck Cancer	1	NCT00858663 Start date: March 2009 Completion date: July 2013
	mTOR	Glioblastoma	1/2	NCT01062399 Start date: December 2010 Completion date: May 2022
	mTOR	Cervix Cancer	1	NCT01217177 Start date: December 2011 Completion date: April 2014
Temsirolimus	mTOR	Rhabdomyosarcoma	3	NCT02567435 Start date: June 2016 Completion date: October 2025
	mTOR	Diffuse Intrinsic Pontine Glioma	1	NCT02420613 Start date: October 2015 Completion date: October 2024
	mTOR	Non-Small-Cell Lung Cancer	1	NCT00796796 Start date: March 2009 Completion date: July 2011
	mTOR	Glioblastoma	1	NCT00316849 Start date: May 2006 Completion date: November 2010
	mTOR	Glioblastoma	2	NCT01019434 Start date: October 2009 Completion date: March 2014

Clinical trial data was obtained from clinicaltrials.gov in May 2025.

## Data Availability

Not applicable.

## Abbreviations

The following abbreviations are used in this manuscript:

4E-BP1	Eukaryotic translation initiation factor 4E-binding protein 1
53BP1	p53-binding protein 1
A2aR	Adenosine A2a receptor
AKT	Protein kinase B
ANXA6	Annexin A6
APLNR	Apelin receptor
ATG5	Autophagy-related gene 5
ATM	Ataxia telangiectasia mutated
ATR	Ataxia telangiectasia and Rad3-related
BABAM1	BRISC and BRCA1-A complex member 1
BAD	Bcl-2-associated agonist of cell death
Bax	Bcl-2-associated X protein
Bcl-2	B-cell lymphoma 2
C2orf40	Chromosome 2 open reading frame 40
CALD1	Caldesmon 1
CD44	Cluster of differentiation 44
CDK1	Cyclin-dependent kinase 1
Chk2	Checkpoint kinase 2
c-Jun	Cellular Jun proto-oncogene
CRC	Colorectal cancer
CSCs	Cancer stem cells
DDRs	DNA damage responses
DEPTOR	DEP domain-containing mTOR-interacting protein
DNA-PKcs	DNA-dependent protein kinase catalytic subunit
DSBs	DNA double-strand breaks
EMT	Epithelial–mesenchymal transition
EpCAM	Epithelial cell adhesion molecule
ESCC	Esophageal squamous cell carcinoma
FA	Fanconi anemia
FAM135B	Family with sequence similarity 135, member B
FANCD2	Fanconi anemia complementation group D2
FOXO1	Forkhead Box O1
GBM	Glioblastoma
Glut-1	Glucose transporter 1
GSK-3β	Glycogen synthase kinase 3 beta
GβL	G-protein β-subunit-like protein
HIF-1α	Hypoxia-inducible factor 1-alpha
HNSCC	Head and neck squamous cell carcinoma
HR	Homologous recombination
Hsp90	Heat shock protein 90
IMRT	Intensity-modulated radiotherapy
LC3-II	Microtubule-associated protein 1 light chain 3 II
MDSC	Myeloid-derived suppressor cell
mLST8	Mammalian lethal with SEC13 protein 8
MRN	MRE11-RAD50-NBS1 complex
mTOR	Mammalian target of rapamycin
Nbs1	Nijmegen breakage syndrome 1 protein
NEDD8	Neural precursor cell expressed, developmentally downregulated 8
NHEJ	Non-homologous end joining
NPC	Nasopharyngeal carcinoma
NSCLC	Non-small-cell lung cancer
OCT-4	Octamer-binding transcription factor 4
OS	Overall survival
P62	Sequestosome-1
PARP1	Poly(ADP-ribose)polymerase 1
PCNA	Proliferating cell nuclear antigen
PDK1	Phosphoinositide-dependent protein kinase 1
PFS	Progression-free survival
PI3K	Phosphatidylinositol 3-Kinase
PRAS40	Proline-rich AKT substrate of 40 kDa
PTEN	Phosphatase and tensin homolog
RAPTOR	Regulatory-associated protein of mTOR
Rad51	RAD51 recombinase
Rb	Retinoblastoma protein
RCC	Renal cell carcinoma
RFC1	Replication factor C subunit 1
RNR	Phosphorylates ribonucleotide reductase
ROS	Reactive oxygen species
S2056	Serine 2056
S6K	Sibosomal protein S6 kinase
SBRT	Stereotactic body radiotherapy
SCLC	Small-cell lung cancer
SIN1	Stress-activated protein kinase-interacting protein 1
SIRT6	Sirtuin 6
SLC7A5	Solute carrier family 7 member A5
SMA	Smooth muscle actin
SOX2	SRY-box transcription factor 2
SSBs	Single-strand breaks
TAMs	Tumor-associated macrophages
TEH	Tenacissoside H
VEGF	Vascular endothelial growth factor
